# Initial Results from the Survey of Organizational Research Climates (SOuRCe) in the U.S. Department of Veterans Affairs Healthcare System

**DOI:** 10.1371/journal.pone.0151571

**Published:** 2016-03-11

**Authors:** Brian C. Martinson, David Nelson, Emily Hagel-Campbell, David Mohr, Martin P. Charns, Ann Bangerter, Carol R. Thrush, Joseph R. Ghilardi, Hanna Bloomfield, Richard Owen, James A. Wells

**Affiliations:** 1 Minneapolis VA Health Care Center, Center for Chronic Disease Outcomes Research, Minneapolis, Minnesota, United States of America; 2 HealthPartners Institute, Bloomington, Minnesota, United States of America; 3 Boston VA, Center for Healthcare Organization and Implementation Research, Boston, Massachusetts, United States of America; 4 University of Arkansas for Medical Sciences, Little Rock, Arkansas, United States of America; 5 Little Rock VA, Center for Mental Healthcare & Outcomes Research, Little Rock, Arkansas, United States of America; 6 James Wells Consulting, Edgerton, Wisconsin, United States of America; Tilburg University, NETHERLANDS

## Abstract

**Background:**

In service to its core mission of improving the health and well-being of veterans, Veterans Affairs (VA) leadership is committed to supporting research best practices in the VA. Recognizing that the behavior of researchers is influenced by the organizational climates in which they work, efforts to assess the integrity of research climates and share such information with research leadership in VA may be one way to support research best practices. The *Survey of Organizational Research Climate* (SOuRCe) is the first validated survey instrument specifically designed to assess the organizational climate of research integrity in academic research organizations. The current study reports on an initiative to use the SOuRCe in VA facilities to characterize the organizational research climates and pilot test the effectiveness of using SOuRCe data as a reporting and feedback intervention tool.

**Methods:**

We administered the SOuRCe using a cross-sectional, online survey, with mailed follow-up to non-responders, of research-engaged employees in the research services of a random selection of 42 VA facilities (e.g., Hospitals/Stations) believed to employ 20 or more research staff. We attained a 51% participation rate, yielding more than 5,200 usable surveys.

**Results:**

We found a general consistency in organizational research climates across a variety of sub-groups in this random sample of research services in the VA. We also observed similar SOuRCe scale score means, relative rankings of these scales and their internal reliability, in this VA-based sample as we have previously documented in more traditional academic research settings. Results also showed more substantial variability in research climate scores *within* than *between* facilities in the VA research service as reflected in meaningful subgroup differences. These findings suggest that the SOuRCe is suitable as an instrument for assessing the research integrity climates in VA and that the tool has similar patterns of results that have been observed in more traditional academic research settings.

**Conclusions:**

The local and specific nature of organizational climates in VA research services, as reflected in variability across sub-groups within individual facilities, has important policy implications. Global, “one-size-fits-all” type initiatives are not likely to yield as much benefit as efforts targeted to specific organizational units or sub-groups and tailored to the specific strengths and weaknesses documented in those locations.

## Introduction and Background

In pursuing its core mission of improving the health and well-being of Veterans, the VA maintains a sizable research service, whose proper functioning is predicated on sustaining the integrity of VA research. In service to these goals, VA leadership is interested in supporting research best practices in the VA. Historically, concern about threats to research integrity have focused primarily on “bad apple” individuals, particularly those who engage in the most egregious forms of misbehavior.[1] More recently, there has been increased recognition of the importance of the “apple barrel” itself; i.e. the workplace, in either fostering or undermining research integrity.[2–6] This broader approach is exemplified by the 2002 U.S. Institute of Medicine (IOM) report, *Integrity in Scientific Research*: *Creating an Environment That Promotes Responsible Conduct*. The IOM report promoted a performance-based, self-regulatory approach to fostering research integrity, recommending that institutions seeking to create sound research climates should: 1) establish and continuously measure their structures, processes, policies, and procedures, 2) evaluate the institutional climate supporting integrity in the conduct of research and 3) use this knowledge for ongoing improvement.[7]

Research integrity has been defined as a complex phenomenon that "characterizes both individual researchers and the institutions in which they work.”[2,7] At the individual level this has been defined as “active adherence to the ethical principles and professional standards essential for the responsible practice of research.”[8] This represents researchers’ commitment to professional norms such as honesty, collegiality, trustworthiness and regard for the accuracy of the scientific record. At the organizational level, research integrity represents a commitment on the part of institutions to promote and foster climates supportive of ethical behavior on the part of its members. This requires both commitment to ethical conduct and support of integrity in research on the part of institutional leaders, and the creation of institutional structures, processes, and policies to ensure appropriate self-monitoring.[9] Combined, these individual traits and institutional characteristics comprise core elements of organizational climates.

### I’ve heard the term “organizational culture,” but what is “organizational climate?”

Organizational *culture* itself is a variously conceptualized and sometimes contentious metaphor. Pettigrew notes, however, that:

“most scholars now agree that organizational culture is a phenomenon that involves beliefs and behavior; exists at a variety of different levels in organizations; and manifests itself in a wide range of features of organizational life such as structures, control and reward systems, symbols, myths, and human resources practices.”(p. 415)[10]

Organizational *climate* can be viewed as “the shared meaning organizational members attach to the events, policies, practices, and procedures they experience and the behaviors they see being rewarded, supported, and expected.”(p. 115)[11] This focus on observable, and manifest aspects of organizations stands in contrast to the concept of organizational culture, which generally refers to more *underlying* values, beliefs and assumptions that guide behaviors of individuals in an organization, but that are not easily observed, measured or reported on.[12] In other words, organizational climate can be thought of as an observable expression of the culture of a place. Common elements between organizational culture and climate include a focus on the *macro view* of an organization, and emphasis on the *context* in which people work and the *sharedness* of experiences over individual differences. Both also emphasize the *meaning* of that context for organizational members and their behaviors and the role of *leaders and leadership* in setting that context; both are considered to be important to *organizational effectiveness*.[11]

The concept of organizational climate is not referencing climate in the meteorological sense of the word. Yet, the two terms are similar in at least one way; in both cases, we would expect that individuals’ behaviors are shaped and influence by their perceptions and experiences of the climates to which they are exposed. Meteorological climate in a Northern location, such as Minnesota, may be associated with activities such as snow-skiing, and ice-skating, while the meteorological climate in a more Southern location such as California may favor activities such as surfing and roller-blading. So too, do organizational climates influence the behaviors of organizational members. This anticipated influence on member behavior is what underlies our interest in organizational climates.

### Importance of Institutional Climates

The conceptual model underpinning the 2002 IOM report referenced above explicitly acknowledges that what happens within organizations is in part a function of the inputs and resources available, but also that the outputs and outcomes are a function of the character of the organization itself.[13] So a focus on the ethical culture and climate points us to focus on the organization structure, processes, policies, and practices. The 2002 IOM report explicitly recognized the role of the local climate–of the lab, the department, the institution–in shaping the behavior of scientists, and acknowledged that these climates can foster or undermine the integrity of behavior. Organizational climate is also fostered and shaped by institutional leaders who are able to exercise some level of control and influence.[14,15] This is not to say that external influences are not also operative. As researchers, we go about conducting our work within our respective organizations, yet we recognize that this work is also influenced by various external factors–structural, policy, financial, political, etc.. [13] Organizational climate can be thought of as a lens, through which those external factors come to influence the research we conduct. Carefully and thoughtfully focused, these lenses can help to bring out our best. If not carefully configured, they can also be damaging, and can undermine best research practices.[16,17]

#### The Survey of Organizational Research Climate (SOuRCe)

Inspired and informed by the 2002 IOM report, *The Survey of Organizational Research Climate* (SOuRCe) is the first validated survey instrument specifically designed to assess the organizational climate of research integrity in academic research settings.[18–20] The SOuRCe is a form of institutional self-assessment, designed to be completed by organizational members directly involved in academic research, to raise awareness among organizational leaders about where their organizational research climates are strong and where they may need improvements. Developed and validated in traditional academic research settings, including academic health centers and large, research-intensive universities in the United States,[18,19] the *SOuRCe* provides seven measures of the climates for research integrity at the overall organizational level and for organizational sub-units (e.g. departments) and sub-groups (e.g. work roles, such as faculty, graduate students, etc.). Further details about the development and validation of the SOuRCe are available elsewhere.[18]

#### Does a “good” organizational climate mean better research practices?

Data available to date to answer questions about the relationships between organizational climate and actual research behaviors are only correlational, so causality cannot be inferred. That said, Crain et al. (2013) documented significant correlations between the SOuRCe measures of organizational climate and self-reported, research-related behaviors of the respondents in their validation sample (roughly n = 1000 academic faculty and postdocs from 40 of the top U.S. academic medical centers).[19] The self-reported behaviors ranged from the ideal up to and including misconduct. Many of the behaviors reported on in that publication would be considered to undermine the quality of research. The Crain et al study defined a composite behavioral outcome called “neglect” that was coded as a binary (0/1) variable, coded as 1 for individuals who reported having engaged in one or more of a range of neglectful research practices in the prior three years. In that sample, more than 46% of respondents endorsed one or more of these four items. In logistic regression models “predicting” neglect, five of the seven SOuRCe domains were significantly associated (p < .001) with self-reported neglect–all at similar magnitudes, and in expected directions.

### Research Objectives and Rationale

The work presented here reflects one part of a larger research project with multiple research objectives, not all of which are addressed in this manuscript. We note here the full set of study objectives to contextualize the portions of this work we present here. The larger study has three objectives, the first of which is to employ the SOuRCe to characterize research integrity climates in VA for the first time. The second objective is to generate detailed feedback reports to research leaders at study-included facilities, as a form of quality improvement (discussed further below) feedback intervention. Finally, the third objective is to conduct a pilot, randomized trial comparing the effectiveness of distributing reports in only written format to a more intensive intervention directed at research leaders that includes both the written feedback report plus intervention phone discussions with members of our study team, reviewing and interpreting reports. In this manuscript, we report descriptive results of the first use of the SOuRCe in the VA, using this instrument to characterize the organizational research climates of VA medical centers (VAMC) in which we are conducting the aforementioned pilot study. We also briefly present information regarding the feedback intervention reports content.

Publications have used the SOuRCe to characterize research integrity climates in traditional academic research settings, including public universities and academic medical centers.[18,20] Never having fielded the SOuRCe in less traditional research settings, one objective of this project was to test whether the instrument performed comparably in this type of setting. The VA differs from more traditional academic research settings in multiple dimensions, including its structure, workforce, mission, and culture. Structurally, the VA is a nationwide entity, with hundreds of distributed facilities, and well over 100,000 employees. The VA is also a Federal department whose activities are subject to different, and possibly a greater number of, regulations and legal requirements than traditional academic settings. The VA has historically been the subject of a great deal of Congressional and other public attention, not all of which has been positive. How organizational leaders have coped with and responded to this attention has shaped the organizational culture. The VA’s mission is focused first and foremost on meeting the health needs of veterans, with research in this context being specifically focused on serving that larger mission, with a strong emphasis on applied research. Partly as a result of these structural and mission-based features of the organization, the culture of the VA includes a long history of quality improvement efforts, many of which have been informed by “reporting and feedback” processes similar to those we are testing in this study.

While the analyses presented in this manuscript are not hypothesis-driven, per se, we did come to this project with some expectations of how the SOuRCe would perform in this new organizational setting. Most generally, we anticipated that the instrument would perform similarly—i.e. yielding similar means and standard deviations—in VA as it had in traditional academic settings. Having seen meaningful variability by work-roles and fields of study in previous work in University-based samples,[20] we also anticipated that we might observe meaningful variation along similar dimensions in the VA sample. Given our intention to use the SOuRCe results in our subsequent quality improvement intervention efforts, we also expected that the tool would yield meaningful differences in scale scores and research integrity climate profiles among the VA facilities included in our study.

Ethics approval was obtained from the VA Central Institutional Review Board and informed consent was provided by each participant, as implied by their completing the anonymous survey, under a waiver of documentation of consent as provided for in 45 CFR 46.117 (c) (2). (CIRB # 13–19).

## Methods

### Study Design and Population Studied

We conducted an online survey with email invitations and mailed follow-up to non-respondents, in which we administered the SOuRCe to research-engaged employees at a random selection of 42 VA facilities (e.g. Hospitals/Stations) with medium to large research services (Min N = 20 such employees, Max N = 600+ such employees). The overall goals of this project required that we sample both individual VA researchers as well as VA facilities.

#### Differential Organizational Receptivity to Quality Improvement Feedback

Quality Improvement, or QI, is a shorthand term describing systematic and ongoing (continuous) efforts and actions aimed at creating measurable improvements in organizational performance.[21] QI is usually considered an iterative process, involving reporting and feedback systems. Organizational theory and empirical evidence tell us that we should expect facilities to vary in their capacity, capability and readiness to make organizational changes.[22–25] We expect that such differences may be related to how receptive and responsive organizational leaders would be to our intervention feedback in this project. Given these expectations, we incorporated in our study design a ranking of facilities by their likely receptivity to QI input. Aside from the study statistician (DN) all team-members are being kept blinded to this ranking until after all follow-up measurements are complete. We based this measure, in part, on expert judgments of two leaders from VA Office of Research and Development, who had pertinent first-hand knowledge of VA Research Service leaders derived from, among other things, having engaged in face-to-face site-visits at these facilities. These leaders were asked to independently rate facilities from Low (-1) to Moderate (0) to High (1) on receptivity and we formed an overall rating by summing these ratings. Recognizing that such expert judgments always retain some subjectivity, we included additional inputs to develop our QI receptivity measure that we felt would be less subjective. Specifically, from the 2011 VA All Employee Survey (AES) and the VA Learning Organizations Survey (LOS) we evaluated broad organizational climates using measures of “Entrepreneurial culture” and “Bureaucratic culture” from the AES, and measures of “Supportive Learning Environment,” “Experimentation”, “Training,” and “Systems Redesign” from the LOS. These measures were selected on input from research team leaders, face validity, and use within the field for improvement purposes by the VA National Center for Organization Development. Using the first principal component for these latter measures, facilities were categorized as having, a) low to moderate values for both the expert-rated QI receptivity scale, and the first principal component or b) moderate to high values for both, with c) the remaining facilities placed in a middle stratum. These categories formed the strata from which we randomly selected VA facilities for inclusion in our sample frame. Overall, among the 95 facilities initially identified for potential inclusion in the study, we had receptivity measures available for 82 facilities with 24 (29%), 35 (42%), and 23 (28%) falling in the lower, middle, and higher QI receptivity strata; these proportions are highly similar to the proportions in each stratum among all classified facilities.

#### Identifying potentially eligible survey participants

To identify potential survey participants we first identified individuals who had completed VA mandatory research training through the Collaborative Institutional Training Initiative (CITI) between May 1^st^, 2011 and October 25^th^, 2013. From these records, in addition to name, we identified individuals’ VA facility of employment, self-reported work role, and email address. Using facility and work role information, we excluded individuals from the four facilities at which study team members worked, individuals from VA administrative offices, individuals with only research regulatory or compliance roles, and individuals with missing location or role information. After further limiting records to those from sites with a minimum of 20 potentially eligible respondents, we identified 38,178 training records. We use the term “potentially eligible” here because being engaged in research in the year prior to the survey was an eligibility requirement that we could not determine with 100% certainty from our sample-frame information alone. Making this determination required the inclusion of a screening question in the survey, and excluding those who indicated they were not engaged in research. We discuss below the implications this had for our process of identifying VA Facilities for study inclusion.

Email records for these individuals were not complete and were often for non-VA email accounts. We merged CITI records with VA Microsoft Exchange Server Global Address List (GAL) records having valid VA emails to identify cases with exactly matching email addresses and either i) exact matches on full name, ii) one name wholly contained in the other name, or iii) exact matches on last name. We identified an additional set of cases with matches on last and first names that also matched on Veterans Integrated Service Network (VISN)/facilities codes, with matching or non-discordant job titles in CITI and GAL. Eliminating those with a non-compensation (WOC) status we used the 20,010 identified cases to identify 95 facilities likely to have at least 20 individuals engaged in research with identifiable VA email addresses. We could classify 82 of these facilities according to the QI receptivity scale described above.

We were concerned that by more strictly matching by name several facilities would be eliminated for lacking the minimum number of identified research staff needed. To refine the coarse matching of CITI and GAL records at this point, we further assessed the likely matches through manual review of the coarse matches incorporating work role information available in both CITI and GAL. However, to reduce the number of records needing to be manually reviewed, we randomly selected an initial subset of 50 facilities (eight more than our targeted number of 42), with 16, 18, and 16 facilities from the low, middle, and high QI-receptivity strata of this scale, respectively.

Further data cleaning and elimination of duplicate records (details available upon request) for these 50 facilities yielded 15,258 likely matches with at least 20 research engaged employees identified for all 50 facilities. The study statistician randomly selected 14 sites from each QI receptivity stratum yielding 42 sites with 12,606 likely research engaged individuals with VA email addresses. The sample size of 42 facilities was chosen based on the feasibility of implementing the survey process in an anticipated sample frame of approximately 12,000 to 14,000 individuals and the feasibility of administering and assessing the study feedback, and implementing an intervention process using that feedback data in a subsequent study step.

### Survey Content

The administered survey included both the SOuRCe and an additional number of classification items allowing us to sub-set the results along potentially salient dimensions. The SOuRCe has been described in detail previously, including its development and validation.[18,20] Briefly, the SOuRCe is a 32-item survey designed to assess an individual’s perception of the organizational climate for research integrity both in one’s general organizational setting and in one’s specific affiliated department, division or work-area. Each SOuRCe item asks respondents to rate the extent to which some quantity or factor is present in their organizational setting, using the same 5-point (Likert) scale: (1) *not at all*, (2) *somewhat*, (3) *moderately*, (4) *very*, and (5) *completely*. Recognizing that some survey respondents may feel they lack adequate experience, exposure or knowledge to answer a given question informedly, each SOuRCe item also includes a “No basis for judging” (NBFJ) response option. In our analyses, we treat NBFJ responses as missing data. The SOuRCe yields 7 scales of organizational research climate: Resources for Responsible Conduct of Research (RCR), Regulatory Quality, Integrity Norms, Integrity Socialization, Departmental Expectations, Supervisor/Supervisee Relations, and Integrity Inhibitors. Scale scores are computed as the average of all items comprising a scale for which a respondent provided a valid (i.e. non-missing) response. For any individual respondent, a scale score is computed only if they provided valid responses to at least half of the items comprising that scale.

In addition to the SOuRCe instrument proper, the survey collected information on work role (faculty/investigator, leadership administrative staff, etc), research area (health services, biomedical, clinical, rehabilitation), length of service in VA (less than 3 years vs. 3+ years), whether the researcher worked exclusively in VA or split their time between VA and non-VA sites, for example an affiliated university, and whether the researcher also had clinical practice duties (yes/no). As noted in the Research Objectives and Rationale section above, inclusion of some of these classification measures was informed by our expectations of where there might be meaningful sub-group variability in SOuRCe scale scores (e.g. by work-role and areas of research). Other classification measures were included as potential additional areas of meaningful variability in SOuRCe scores (e.g. length of service, having clinical responsibilities in addition to research role, and whether one worked exclusively in the VA).

### Survey Implementation

Survey fielding began with a notification email, sent to all individuals thought to be engaged in research at the sampled facilities with an identified email address. This notice was clearly demarcated as coming from within the VA itself, including contact information for the Project Coordinator, was signed by the study Principal Investigator (BCM), indicated our plan to send a survey invitation within several days, and providing brief information on the nature of the survey. This was followed several days later by the initial invitation email, including a hyperlink to the survey website, which was accessible only from within the VA firewall. The survey webpage included participant instructions, informed consent statement, and links to the survey itself. Participants were assured that their survey responses would be collected completely anonymously, with no way to connect their responses back to them as individuals, and informed that their consent to participate would be indicated by clicking on the link to the web-based survey. We informed potential participants that to protect their identities we would not release any individual-level data, and that only “aggregate statistics [would] be reported.” We have followed the VA standard for such aggregation of requiring a minimum of ten individuals in a group for data on that group to be reported. This reporting restriction was incorporated in the initial research protocol and IRB approval process. A tool on this website was used to keep track of respondents and completed surveys. Up to four reminder emails, also including hyperlinks to the survey website, were sent at roughly four business-day intervals to non-respondents, with email batches originating on different days of the week. Non-respondents to the email recruitment campaign (n = 7,477) were sent a paper copy of the survey via USPS mailing, with the packet including a cover-letter including survey instructions and informed consent statement, and a self-addressed, metered, return envelope. The survey website remained “open” throughout the survey fielding process, and those mailed paper surveys were informed they could complete the survey on paper, or by visiting the survey URL that was printed on the face-page of the paper survey.

### Statistical Analysis

Our objective of using the SOuRCe to characterize the organizational research climates in VA facilities leads us to present primarily descriptive data here. We consider the average SOuRCe scales within facilities as characterizing the facility research climates. All SOuRCe items are scored from 1 to 5 and the SOuRce scale scores constitute the average of scores for all non-missing items contributing to the scale. We initially constructed descriptive results for each scale at the individual level to compare SOuRCe scale ratings in the VA to those from previous studies and used Cronbach’s alpha to assess the reliability of the SOuRCe scales in this population. We also examined whether facility average scale scores varied across QI-receptivity strata, using analysis of variance, and whether survey response rates were correlated with the observed facility average climate scores to assess possible sampling and response biases.

We also wanted to assess whether perceived climate varied across key subgroups comprising research area, research role, and other individual characteristics. For this aim, in addition to summarizing the distribution of facility average subgroup scales, we used generalized linear mixed models (GLMMs) in which each SOuRCe scale was regressed individually on one classification item at a time. All GLMM models include random intercepts for facility and individual-level error terms. We used the model Wald test to assess whether scales scores differed by subgroups, using a significance level of 0.01; for those scale scores with significant test results we identified significant pairwise differences. Given the current state of knowledge, we lack a gold-standard criterion on which to judge how large a difference in predicted mean scores is “substantively meaningful.” In the absence of such a criterion, we focus attention only on mean differences between sub-groups that are at least 0.20 in absolute value. Although this is a somewhat arbitrary threshold, it represents an absolute difference roughly one-quarter of an individual level standard deviation, so represents a “small to moderate,” effect size, and approximately one facility level standard deviation.

## Results

We fielded the SOuRCe from February to April 2014 with email invitations sent to the 12,606 identified research-engaged VA employees. Among these, 2,366 were unreachable (no longer at VA, email inbox full, etc.), and 83 were determined ineligible (having moved to a different VA facility, etc.), yielding a potential sample size of N = 10,157. Among these, 77 actively declined and 5,225 responded with at least partially completed surveys (sufficient to calculate at least one climate scale). Thus, our survey participation rate was 51.4%.

The size of the identified research service groups ranges broadly across the sampled facilities, with the smallest comprising 29 individuals and the largest 696 (Median of 259). Participation rates across facilities ranged from a low of 29% to a high of 92% (Mean of 54%). One facility was excluded due to having fewer than 10 usable responses. These factors combined resulted in a total of 4,221 observations from 41 facilities usable for analysis and reporting, varying from a minimum of 16 to a maximum of 314 (median of 94) per facility.

**Table 1** presents the number of usable responses, number of responses missing due to NBFJ responses, means, standard deviations and reliability statistics (Cronbach’s alpha) for each of the seven SOuRCe scales. The number of usable responses varies across climate scales, due to item-missingness, which results from items being unanswered, or individuals selecting a NBFJ option for individual scale items. For all scales, the majority of missing cases resulted from respondents selecting the NBFJ response option to more than half of the items in the scale. This ranged from 56% of the cases missing for the Integrity Norms scale to a high of 98% of the cases missing for the Regulatory Quality scale. With the exception of the mean score for the integrity inhibitors scale, all scale means are somewhat higher in this sample than they were in the two prior (non-VA) samples for which there is published data.[18,20] These generally higher means notwithstanding, the relative “ranking” of scale means is similar to what we have observed in more traditional academic settings—with the highest mean being observed for the Integrity Norms scale (4.45), the lowest mean being observed for the Integrity Socialization scale (3.86), and the remaining five scales falling between these two extremes, in descending order: Supervisor/Supervisee Relations (4.11), RCR Resources (4.05), Departmental Expectations and Regulatory Quality (both at 4.02), and Absence of Integrity Inhibitors (3.89). The reliability of scales in this sample, as assessed by Cronbach’s alpha, are also quite comparable to what we have observed in other settings–in this sample ranging from 0.80 for the Departmental Expectations scale, to 0.87 for several other scales.

**Table 1:** Number of Cases, Means, Standard Deviations, and Reliability by Climate Scale.

	Departmental Expectations	Integrity Norms	Integrity Socialization	Absence of Integrity Inhibitors	RCR Resources	Regulatory Quality	Supervisor/ Supervisee Relations
**n usable**	3631	3964	3852	3712	4175	3795	3712
**n NBFJ missing**	529	145	299	433	30	417	371
**M**	4.02	4.45	3.86	3.89	4.05	4.02	4.11
**SD**	0.86	0.60	0.93	0.88	0.80	0.87	0.83
**n of items**	2	4	4	6	6	3	3
**Reliability(α)**	0.80	0.81	0.86	0.82	0.87	0.87	0.87

Notes: RCR = Responsible Conduct of Research

### Correlations between facility-level participation rates and SOuRCe scale scores

The wide-range of participation rates across the facilities in our sample led us to examine whether this variability may have influenced our results. We explored this by examining the correlation, at the facility level, between participation rate and scale scores, which we present in Table 2.

**Table 2:** Correlations between facility means and facility participation rates.

	Correlation	P value
**Departmental Expectations**	-0.13	0.43
**Integrity Norms**	-0.16	0.31
**Integrity Socialization**	-0.21	0.18
**Absence of Integrity Inhibitors**	-0.11	0.51
**RCR Resources**	-0.09	0.58
**Regulatory Quality**	0.02	0.91
**Supervisor/ Supervisee Relations**	-0.38	0.01

For most scales, we observed only modest, negative correlations that were not statistically significant. One notable exception to the pattern of modest negative correlations is a moderately strong, and statistically significant negative correlation (-0.38, p = 0.01) for the supervisor/supervisee relations scale. Subsequent exploration revealed that this correlation is driven by one influential facility with a high participation rate (83.3%) and a very low, outlying, mean for the supervisor/supervisee relations scale (3.33). Exclusion of this one facility from the correlation analysis results in a non-significant correlation between participation rate and supervisor/supervisee relations very much in line with the other scales (-0.18, p = 0.28). Correlations here do not necessarily indicate non-response bias in the facility level measure because we might expect higher participation rates from sites with both less favorable climates as well as from those with more favorable climates. However, our concerns about the potential presence of non-response bias at the individual level are reduced by the observation of generally small and non-significant correlations here.

### SOuRCe scale means by assessed receptivity to quality-improvement input

We assessed whether facility average SOuRCe scales varied across QI receptivity strata using generalized linear mixed model regressions in which each SOuRCe scale was regressed individually on the QI-receptivity measure, coded as “low,” “moderate,” or “high.” For six of the seven scales, these models yielded only small differences in predicted scale means across the QI-receptivity categories, and non-significant Type III Tests of these fixed effects. One exception to this was for the Absence of Integrity Inhibitors scale, which yielded a significant Type III test (p = 0.021) and higher predicted means for both the low receptivity group (μ = 3.91, SD = 0.20), and the high receptivity group (μ = 3.94, SD = 0.14), compared to the moderate receptivity group (μ = 3.76, SD = 0.25).

### Variation in facility average SOuRCe scales across VA research organizations

In Table 3 we present percentile distributions, mean, minima and maxima, and measures of variability of the organizational climate scales across the 41 sampled facilities using facility as the unit of analysis and facility average scale scores as the facility level measures of research climate. The average and median facility-level scale scores are similar to the individual-level scale mean scores seen in Table 1. Moreover, as seen in the relatively tight interquartile range for each scale in Table 3, we observed only modest variability at the level of VA facility. These high and consistent means across facilities, combined with the fairly high observed minima indicate that, in the aggregate, VA research services had generally favorable climates, at least during the period about which we asked respondents to report (roughly the year prior to May 2014).

**Table 3:** Percentile Distributions and Variability of Means Climate Scale Scores by Facility.

	Departmental Expectations	Integrity Norms	Integrity Socialization	Absence of Integrity Inhibitors	RCR Resources	Regulatory Quality	Supervisor/ Supervisee Relations
**n of facilities**	41	41	41	41	41	41	41
**Distribution**							
Maximum	4.43	4.65	4.13	4.43	4.27	4.50	4.33
75^th^ %tile	4.07	4.51	3.91	3.99	4.12	4.18	4.18
Mean	3.98	4.44	3.78	3.87	3.99	4.03	4.07
Median	4.01	4.43	3.82	3.87	4.01	4.04	4.09
25^th^ %tile	3.89	4.37	3.69	3.76	3.88	3.88	4.00
Minimum	3.66	4.25	3.34	3.31	3.57	3.48	3.33
**Variation**							
Interquartile	0.17	0.13	0.22	0.23	0.24	0.30	0.18
SD	0.18	0.10	0.19	0.21	0.18	0.22	0.17

Recall that our facility sampling strategy somewhat under-represented facilities from the moderate QI receptivity stratum (we selected equal numbers of facilities from each stratum, but a higher proportion of all facilities fell into the moderate stratum than into either the high or low strata). This fact, combined with the potential variation across the QI receptivity strata in the Absence of Integrity Inhibitors scale means the summary measures presented in Table 3 may not completely reflect the distribution of these scales across all VA research organizations. We constructed sample design weighted estimates of the means and percentiles of the distribution of the average SOuRCe scales across VA research organizations using weights based on the stratified sampling of the facilities from the initial 82 stratified facilities. Results differed from those presented in Table 3 only in the second decimal.

### SOuRCe Scale means by sample sub-groups

Tables 4 and 5 display results of two GLMM regressions in which each SOuRCe scale is regressed individually on work role (4) and primary research service (4) in separate models. In both panels, the left-most column identifies the scale, the F test statistic value and P value for a given regression model (assessed by Type III sums of squares). **Table 4** displays regression results by respondent work role–where role categories include Investigator or Faculty (47% of respondents), Leadership or Administrative Staff (7%), Research Support Staff (34%), Students & Fellows (8%; including graduate students and postdocs), and an un-specified “Other” category (4%) encompassing respondents who could not be classified into one of the preceding work role categories. In this panel, four climate scales display what we consider to be meaningful differences in the absolute size of predicted means between groups: Departmental Expectations, Integrity Socialization, Regulatory Quality and Supervisor/Supervisee Relations. In each of these four models, the predicted means for Research Support Staff, Students/Fellows, or both, are meaningfully higher than the predicted means for Investigators/Faculty. For the Regulatory Quality scale, respondents who were in Leadership or Administrative Staff roles also report a higher mean score than do Investigators/Faculty. For the Supervisor/Supervisee Relations scale the Leadership/Administrative Staff mean score is significantly lower than the mean score for Students/Fellows. Our conclusion is that, to some extent, work role differences between Investigators/Faculty and other research staff present a meaningful source of variability in reported organizational climates in VA research services.

**Table 4:** Generalized Linear Mixed Model Regressions of SOURCE scales by respondent characteristic of work-role.

Work Role	Investigators/Faculty (REF.) (N~1792)		Leadership or Administrative Staff (N~275)		Research Support Staff (N~1296)		Student/Fellow(N~313)		Other (N~158)	
Scale F (Pvalue, df)	Beta (SE)	Pred. Mean	Beta (SE)	Pred. Mean	Beta (SE)	Pred. Mean	Beta (SE)	Pred. Mean	Beta (SE)	Pred. Mean
**Departmental Expectations** 13.42 (<0.0001, 3586)	-	**3.91**^**a**^	0.17 (0.06)	4.08	0.22 (0.03)	**4.13**^**b**^	0.13 (0.05)	4.04	-0.03 (0.07)	3.87
**Integrity Norms** 1.83 (0.12, 3919)	-	4.43	0.02 (0.04)	4.45	0.05 (0.02)	4.47	0.03 (0.04)	4.46	-0.05 (0.05)	4.37
**Integrity Socialization** 16.8 (<0.0001, 3807)	-	**3.71**^**a**^	0.16 (0.06)	3.87	0.23 (0.03)	**3.94**^**b**^	0.27 (0.06)	**3.98**^**b**^	-0.09 (0.07)	3.62
**Absence of Integrity Inhibitors** 2.32 (0.05, 3667)	-	3.90	-0.02 (0.06)	3.88	-0.03 (0.03)	3.87	-0.03 (0.05)	3.87	-0.22 (0.07)	3.68
**RCR Resources** 2.65 (0.03, 4130)	-	3.97	0.08 (0.05)	4.05	0.07 (0.03)	4.04	0.09 (0.05)	4.07	-0.03 (0.06)	3.94
**Regulatory Quality** 15.61 (<0.0001, 3750)	-	**3.92**^**a**^	0.23 (0.05)	**4.15**^**b**^	0.23 (0.03)	**4.14**^**b**^	0.18 (0.05)	4.10	0.09 (0.07)	4.00
**Supervisor/ Supervisee Relations** 5.85 (0.0001, 3667)	-	**4.05**^**a**^	-0.02 (0.06)	**4.03**^**a**^	0.08 (0.03)	4.13	0.22 (0.05)	**4.27**^**b**^	-0.002 (0.07)	4.05

Boldfaced cells indicate statistically significant F-values and meaningful differences in predicted means where those demarcated with an “a” are different from those demarcated with a “b.”

**Table 5:** Generalized Linear Mixed Model Regressions of SOURCE scales by respondent characteristic of primary research area.

Area of Research	Clinical (REF.) (N~2065)		Biomedical (N~380)		Health Services (N~840)		Rehabilitation (N~311)		Other (N~230)	
Scale F (P-value, df)	Beta (SE)	Pred. Mean	Beta (SE)	Pred. Mean	Beta (SE)	Pred. Mean	Beta (SE)	Pred. Mean	Beta (SE)	Pred. Mean
**Departmental Expectations** 4.30 (0.002, 3579)	-	3.95	0.05 (0.05)	4.00	0.13 (0.04)	4.09	0.15 (0.05)	4.10	0.07 (0.06)	4.02
**Integrity Norms** 1.79 (0.13, 3911)	-	4.44	-0.03 (0.03)	4.41	0.02 (0.02)	4.46	0.08 (0.04)	4.52	-0.03 (0.04)	4.41
**Integrity Socialization** 3.64 (0.006, 3800)	-	3.78	0.02 (0.05)	3.80	0.09 (0.04)	3.87	0.17 (0.06)	3.94	0.13 (0.06)	3.91
**Absence of Integrity Inhibitors** 5.40 (0.0002, 3660)	-	3.84	0.01 (0.05)	3.85	0.12 (0.04)	3.96	0.17 (0.05)	4.01	-0.09 (0.06)	3.75
**RCR Resources** 4.44 (0.001, 4122)	-	3.97	0.05 (0.04)	4.02	0.11 (0.03)	4.08	0.13 (0.05)	4.10	0.06 (0.05)	4.03
**Regulatory Quality** 2.28 (0.06, 3743)	-	4.00	0.02 (0.05)	4.03	0.06 (0.04)	4.06	-0.04 (0.05)	3.96	0.15 (0.06)	4.15
**Supervisor/ Supervisee Relations** 1.04 (0.39, 3659)	-	4.08	0.04 (0.05)	4.12	0.04 (0.04)	4.12	0.09 (0.05)	4.17	-0.02 (0.06)	4.07

**Table 5** displays regression results by respondents’ primary research area–defined as Biomedical (10% of respondents), Clinical (54%), Health Services Research (22%), Rehabilitation research (8%) and an un-specified “Other” category (6%) which represented a small number of Veterinary research respondents, and respondents who could not be otherwise classified. In most, but not all instances, predicted means are slightly lower in the Clinical research group than in the other groups, and highest in the Rehabilitation Research group. Regressions in this panel with statistically significant values include those for Departmental Expectations, Integrity Socialization, Absence of Integrity Inhibitors, and RCR Resources. However, none of the absolute differences between predicted means in this panel reach our threshold of 0.20, although the area of Rehabilitation Research comes the closest to having means that are meaningfully higher than those for clinical researchers for each of the seven scales, and the Biomedical and Health Services areas fall between these other two groups. Although there are some differences in climate scale means by research area, in general these differences may not constitute a meaningful source of variability in reported climate scales in VA research service.

### Scale means by other respondent characteristics

We explored whether significant variability exists in SOuRCe scale scores by several individual-level characteristics. These included duration of employment in VA contrasting those employed in VA for two years or less (36%) with those employed for three or more years (64%), whether respondents had clinical care responsibilities (60%), and whether respondents conducted research exclusively in VA (75%) or at both VA and elsewhere, such as at an affiliate university.

GLMM regressions of the SOuRCe scale scores by each of these characteristics yielded statistically significant F values for various scales, however, none of these models yielded absolute differences between predicted mean scores that reached our substantively meaningful threshold of 0.20.

### SOuRCe scale means by affiliation with a research Center of Excellence

For some areas of research in VA, including both Rehabilitation and Health Services Research, there are Centers of Excellence (COE) housed at select facilities. These COEs provide an organizational structure for those affiliated with them that is absent in places without these centers, and which may be an important contributor to the integrity of local research climates. To assess this possibility, using ancillary administrative information, we classified respondents in our sample as being affiliated with COEs based on a combination of their self-reported area of research and available information about COEs at each facility. We presumed that an individual who reported their area as a Rehabilitation Researcher and who was located at a facility with rehabilitation research COE was affiliated with that center, with a parallel assumption being applied to Health Services Researchers.

Response rates were very similar between facilities with and without COEs, differing by less than two percentage points. Among N = 339 Rehabilitation Researchers, 205 (60%) were employed at 10 facilities with a Rehabilitation COE. For six of the seven SOuRCe scales, we observed very similar means between respondents who were and were not affiliated with a Rehabilitation COE. Only for the regulatory quality scale, did we observe a difference that we deem substantively meaningful, with center-affiliated rehabilitation researchers having lower mean scores (μ = 3.8, SD = 0.9) than those at facilities without such a center (μ = 4.1, SD = 0.8).

Of the 928 health services researchers, 578 (62%) were employed at 10 facilities with a Health Services Research and Development (HSR&D) COE. For all seven SOuRCe scales, means were either similar or were higher for center-affiliated HSR&D researchers, compared to HSR&D researchers at facilities without such a center. For three scales, these differences were of a magnitude that we would deem substantively meaningful. For center-affiliated HSR&D researchers, the Departmental Expectations scale mean was 4.2 (SD = 0.8) compared to 4.0 (SD = 0.9) for those not center-affiliated. The Integrity Socialization scale mean was 4.0 (SD = 0.9) compared to 3.8 (SD = 1.0), respectively, and the Absence of Integrity Inhibitors scale was 4.1 (SD = 0.8) compared to 3.8 (SD = 0.9). Our conclusion from these analyses is that for HSR&D researchers, but not for Rehabilitation researchers, being affiliated with a COE is associated with somewhat more favorable perceptions of research climates in VA. Comparing the frequency of NBFJ responses between respondents at facilities with and without COEs, there were generally only small differences (two percentage points or less). The one exception to this was for the Supervisor/Supervisee Relations scale, on which close to 13% of respondents at facilities without COEs were missing due to NBFJ responses, whereas this was true for only about 8% of respondents at facilities with COEs (Chi-sq p < .0001). We speculate that this may be due to there being more structured research supervision at sites with COEs.

### Within-facility variability in SOuRCe scale means

**Fig 1:**
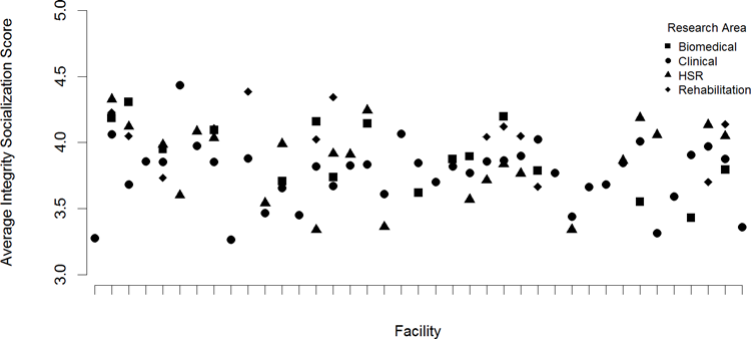
Within Facility Variability in Integrity Socialization Scale, by Area of Research. Research Areas: Square = Biomedical Circle = Clinical Triangle = HSR Diamond = Rehabilitation. Note: Observations for two facilities omitted due to insufficient group size to report.

Up to this point, we have examined variability in SOuRCe scale scores across facilities by aggregating our data at the facility level or by looking for consistent differences across facilities between salient subgroups. The ultimate purpose of collecting the SOuRCe data was to summarize and report these scale scores, along with comparative, aggregate-level scores, to research leaders at individual institutions (in this case, research services at individual VA facilities). By providing these leaders salient sub-group breakouts, we aimed to help them identify, with greater specificity, signs of strengths and weaknesses in their local research climates. By providing the comparative data, we aimed to inform and motivate those with relatively low climate scores to attempt organizational improvement efforts. For the intervention phase of this study, we prepared and returned facility-specific reports to research leaders at each of the 41 facilities in our sample. The details of this fine-grained look at the SOuRCe data are too voluminous to present here; the facility reports ranged from about 20 pages to more than 90 pages each, depending on the size and diversity of the local research service. To give some sense of these details, in Fig 1, we present facility-specific mean scores for a single SOuRCe scale (Integrity Socialization) broken out by a single sub-group dimension (Area of Research).

Mean scores by area of research are plotted on the Y-axis, with 39 observations, one per facility, plotted along the X-axis (two facilities were omitted as none of the research subgroups at these two facilities had a minimum of ten respondents when disaggregating by area of research). In this figure, we observe some facilities where mean-scores cluster tightly (such as the 2^nd^ facility from the left) indicating all sub-areas of research rate this dimension of their local climate similarly, and in this instance highly. By contrast, the overall impression from this figure is one of much more disparate scoring of climates within facilities, across different areas of research, reflected in the numerous facilities in which there is much greater range in mean-scores between research areas. Consistent with what we observed in Table 5, those in clinical research areas tend to report relatively lower scores than other areas of research, but not consistently so. There is substantial variation across sites in the relative ordering of the climate ratings across research areas and in the within facility variation in ratings by the research areas. Our conclusion from this analysis is that there is more meaningful variability in research climate scores *within* than *between* facilities in the VA research service.

## Discussion and Conclusions

Our findings indicate that in terms of SOuRCe scale score means and the relative rankings of these scales, and the internal reliability measures for the scales, research climates in VA appear quite similar in the aggregate to those we have previously documented in more traditional academic research settings. With some exceptions, this study also documents consistency in organizational research climates across a variety of sub-groups in our random sample of research services in the VA. We also obtained a similar level of respondent cooperation in the VA research setting as we have obtained in other settings. Taken as a whole, these findings indicate that the SOuRCe is as suitable an instrument for assessing the research integrity climates in VA as it has proven to be in more traditional academic research settings.

Some limitations of this study warrant mention. Predominantly via inclusion of a screening question included in our survey, we discovered that 15% of respondents at each facility were not engaged in research, reflecting limitations of our sample frame. A 51% participation rate leaves room for concern about the potential for non-response bias in our data, if those who choose not to respond are systematically different from those who did, with respect to our SOuRCe measures. We found that facilities with higher participation rates had modestly lower observed mean scores on most SOuRCe scales, which could indicate that survey respondents held slightly less favorable perceptions of these climate scales than did non-respondents, in which case differential non-response might have led to a modest downward bias on most climate scales. Another limitation of this study is our general lack of behavioral outcome criteria against which to judge the climate assessments. While we have previously documented significant correlations between SOuRCe climate scores and self-reported, research-related behavior, concerns about the sensitivity of obtaining such self-report data in the VA led us to avoid including those measures in our data collection.

These limitations notwithstanding, our findings have at least one important policy implication. As seen in Fig 1, there is likely more meaningful variability in research climate scores *within* than *between* facilities in the VA research service. In the aggregate, the integrity of research climates in VA looks to be on par with the integrity of research climates in more traditional academic research settings. So the question is not whether the VA as a whole, or whether a particular VA facility, has a “good” or a “bad” research climate. The local and specific nature of organizational climates, as reflected in variability across sub-groups within individual facilities, suggests that, in terms of efforts to foster and sustain research integrity, there is more to be gained through efforts targeted to specific organizational units or sub-groups and tailored to the specific weaknesses documented than may be gained through the more typical policy application of global, “one-size-fits-all” type initiatives.

The local, specific, comparative results provide by the SOuRCe are what make it particularly useful for informing organizational leaders about the specific strengths and weaknesses in their integrity climates. A couple of specific examples may help to illustrate. At one facility in our sample, our report to leaders revealed a mean score reported by investigator/faculty respondents for the “absence of integrity inhibitors” scale of 3.64, compared to a grand-mean score of 3.86 reported by investigator/faculty respondents overall. This absolute, comparative difference of -0.22 represented a local score roughly one-quarter of a standard deviation lower than the grand-mean, a decrement we considered worthy of leadership concern. By contrast, research support staff in this same facility reported a mean score for the “integrity socialization” scale of 4.17, compared to a grand mean of 3.90 for this same scale among all research support staff–an absolute comparative difference of +0.27. We considered favorable variances of this magnitude relative to their respective grand sample-means as signs of particular strengths in local climates. Assessing local scale scores relative to the overall scale means by definition is benchmarking against only the “average” for the sample, and it could be argued that a standard defining “excellence” might be preferred. While nothing would prevent the use of other such standards for comparisons, our chosen benchmark is suitable for identifying particularly high and particularly low integrity climates. We intentionally did not conduct or present statistical tests of the magnitude of these comparative differences in our reports to research leaders. In addition to the scale mean scores, our reports to research leaders included item-level details that in many cases provide even more targeted information to help guide improvement efforts.

Our results also indicated that individual-differences in duration of employment in VA, whether one has clinical duties in addition to research duties, and whether one conducts research exclusively in VA are not significant predictors of variability in organizational climates in VA research service. One dimension along which we observed substantively meaningful differences in SOuRCe scale scores was work role. We have previously examined variability in SOuRCe scores by work role, in a large sample of respondents pooled across several “Big Ten” universities,[20] though in that previous study, a more limited set of work roles were included than in the current study (e.g. that study did not include leadership or administrative staff or research support staff as separable sub-groups). The overall pattern we observe here is that, relative to other research staff, VA Investigators/Faculty provide lower assessments of multiple dimensions of their research climates. For the Departmental Expectations scale, the observed differences may have something to do with the fact that both scale items ask about productivity expectations that are largely specific to investigators or faculty. Similarly, it may be that the somewhat higher scores on the Regulatory Quality scale reported by those other than Investigators/Faculty may have something to do with role-related differences in how non-investigator staff in VA research interact with regulatory oversight bodies.

Ensuring research integrity and best practices requires attending to the integrity of the research climates in the VA, as elsewhere. Given ever-pressing resource constraints, it is imperative that this be done in a way that reflects good stewardship of public resources. The SOuRCe offers a meaningful method for generating the local and specific information about research climates that can be used to efficiently develop organizational change efforts that are appropriately tailored and targeted. We anticipate that such informed efforts are more likely to achieve the necessary “buy-in” needed to accomplish meaningful improvements than less well-informed initiatives.
